# Genomic prediction of breeding values for carcass traits in Nellore cattle

**DOI:** 10.1186/s12711-016-0188-y

**Published:** 2016-01-29

**Authors:** Gerardo A. Fernandes Júnior, Guilherme J. M. Rosa, Bruno D. Valente, Roberto Carvalheiro, Fernando Baldi, Diogo A. Garcia, Daniel G. M. Gordo, Rafael Espigolan, Luciana Takada, Rafael L. Tonussi, Willian B. F. de Andrade, Ana F. B. Magalhães, Luis A. L. Chardulo, Humberto Tonhati, Lucia G. de Albuquerque

**Affiliations:** Faculdade de Ciências Agrárias e Veterinárias, UNESP, Jaboticabal, SP 14884-900 Brazil; Department of Animal Sciences, University of Wisconsin-Madison, Madison, WI 53706 USA; Faculdade de Medicina Veterinária e Zootecnia, UNESP, Botucatu, SP 18618-970 Brazil

## Abstract

**Background:**

The objective of this study was to evaluate the accuracy of genomic predictions for rib eye area (REA), backfat thickness (BFT), and hot carcass weight (HCW) in Nellore beef cattle from Brazilian commercial herds using different prediction models.

**Methods:**

Phenotypic data from 1756 Nellore steers from ten commercial herds in Brazil were used. Animals were offspring of 294 sires and 1546 dams, reared on pasture, feedlot finished, and slaughtered at approximately 2 years of age. All animals were genotyped using a 777k Illumina Bovine HD SNP chip. Accuracy of genomic predictions of breeding values was evaluated by using a 5-fold cross-validation scheme and considering three models: Bayesian ridge regression (BRR), Bayes C (BC) and Bayesian Lasso (BL), and two types of response variables: traditional estimated breeding value (EBV), and phenotype adjusted for fixed effects (Y*).

**Results:**

The prediction accuracies achieved with the BRR model were equal to 0.25 (BFT), 0.33 (HCW) and 0.36 (REA) when EBV was used as response variable, and 0.21 (BFT), 0.37 (HCW) and 0.46 (REA) when using Y*. Results obtained with the BC and BL models were similar. Accuracies increased for traits with a higher heritability, and using Y* instead of EBV as response variable resulted in higher accuracy when heritability was higher.

**Conclusions:**

Our results indicate that the accuracy of genomic prediction of carcass traits in Nellore cattle is moderate to high. Prediction of genomic breeding values from adjusted phenotypes Y* was more accurate than from EBV, especially for highly heritable traits. The three models considered (BRR, BC and BL) led to similar predictive abilities and, thus, either one could be used to implement genomic prediction for carcass traits in Nellore cattle.

## Background

The Brazilian beef cattle industry is mainly based on purebred and crossbred pasture-fed Zebu animals. Nellore is the predominant beef cattle breed in Brazil, which makes it an important breed in the world beef market [1]. In this context, carcass traits are of paramount importance. Typically, producers are remunerated by cold-storage plants on the basis of kilograms of carcass produced. Cold-storage facilities prefer carcasses with a high muscle/bone ratio and adequate finishing, which are commonly analyzed by rib eye area and fat coverage characteristics, respectively [2].

Although genetic evaluations of carcass traits recorded *post*-*mortem* are important to improve Nellore cattle, routine measurements of these traits are difficult and/or expensive to obtain. Therefore, estimating genetic merit of young animals becomes challenging, especially when using traditional pedigree-based methods, and breeding programs usually do not include these traits formally in their breeding goals [1]. Genomic selection could be an alternative method to achieve accurate genetic evaluations, which, in particular may, help contribute to genetic improvement for carcass traits.

Genomic selection has revolutionized animal breeding by enabling the evaluation of animals based on genome-wide single nucleotide polymorphism (SNP) data [3–6]. Genomic selection exploits the linkage disequilibrium (LD) between SNPs and quantitative trait loci (QTL) [7]. A sufficiently dense panel of SNPs that cover the entire genome is used for this purpose, under the expectation that all QTL are in LD with at least one SNP [5, 8]. Regression of phenotype on SNP genotypes is performed to capture the genetic effect of QTL. Accordingly, the sum of the estimated effects of all SNP genotypes of a selection candidate, i.e. the direct genomic value (DGV), can be interpreted as a predictor of its breeding value [9, 10].

Decisions on which model should be used for genomic prediction are key steps in the implementation of genomic selection. The SNP-based regression models that are commonly used for genomic prediction differ mainly in terms of the assumptions used for the prior distribution of genetic effects. Most proposed priors vary from independent Gaussian distributions [as in Bayesian ridge regression (BRR)] to thick-tailed distributions [as in Bayes A or Bayesian Lasso (BL)] and finite mixtures [as in Bayes C (BC)], which can be modeled as scale mixtures of normal distributions [11]. A comprehensive review and comparison of these models are available in [12]. Traditional (pedigree-based) estimated breeding values (EBV), deregressed EBV (dEBV) and phenotypes adjusted for fixed effects, are pseudo-phenotypes that are commonly used to fit and evaluate prediction models [9, 10, 13–15].

Although the *post*-*mortem* measurement of carcass traits in Nellore cattle is economically important, to date, implementation of genomic selection for these traits has not been investigated for this breed. The objective of this study was to compare the accuracy of genomic predictions obtained with different models and pseudo-phenotypes for hot carcass weight, rib eye area and back fat thickness in Nellore cattle.

## Methods

### Phenotypic and genotypic data file

Phenotypic and genotypic data from 1756 Nellore steers from commercial herds that are located in the southeast, mid-west and northeast of Brazil were used. These animals were offspring of 294 sires and 1546 dams, from ten farms and three different breeding programs (DeltaGen, Paint and Nelore Qualitas). The animals were reared on pasture, feedlot finished, and slaughtered when they were approximately 2 years old (731.9 ± 83.0 days).

The following traits were analyzed: hot carcass weight (HCW), rib eye area (REA), and backfat thickness (BFT). REA and BFT were measured over the *longissimus dorsi* muscle between the 12th and 13th rib of the left half-carcass, according to the method proposed by the United States Standards for Grades of Carcass Beef [16]. Observations that were outside the interval between 3.5 standard deviations above and below the mean of the contemporary group (CG) were removed. Each CG contained at least three animals, and was defined by combinations of year, farm of birth, and management group at yearling. There were 141 CG for REA and BFT and 131 for HCW, with an average of 12 animals in each group. Phenotypic averages were equal to 68.6 ± 8.59 cm^2^, 4.84 ± 2.59 mm and 277.9 ± 23.4 kg for REA, BFT and HCW, respectively.

Tissue samples were collected from the same muscle (*longissimus dorsi*) and were genotyped using a panel of 777,962 SNPs (Illumina Bovine HD chip). Quality control of genotypes was performed using an iterative method. Only autosomal SNPs with a GenCall score greater than 0.70 were considered. Fifty-four pairs of SNPs were excluded because the SNPs of each pair were assigned to the same genomic position. SNPs with a minor allele frequency (MAF) less than 0.02, a Hardy–Weinberg equilibrium *p* value less than 10^−5^, and a call rate less than 0.98 were excluded. Finally, pair-wise correlations (r^2^) between SNPs within 100-SNP windows were estimated and the SNP with the lowest MAF of each highly correlated pair (r^2^ ≥ 0.995) was excluded. Individuals with a call rate less than 0.90 were excluded as well. The process was iterated until no further SNP or individual was excluded. After phenotypic and genotypic editing, the final dataset included 1567 animals and 369,776 SNPs for REA, 1566 animals and 369,835 SNPs for BFT, and 1409 animals and 362,120 SNPs for HCW.

Since animals originated from three breeding programs, a principal component analysis (PCA) based on the genomic relationship matrix (**G**) produced according to [17] was performed to check for population stratification. Results did not indicate the existence of subgroups among the individuals from different breeding programs (data not shown).

### Response variables used for genomic predictions

According to [18] when individual phenotypic and genotypic data are available, joint inference for all effects (fixed and random, including SNP effects) is preferable over genomic predictions using data pre-adjusted for non-genetic effects. However, the number of observations per CG was not sufficiently large to be able to estimate all fixed effects for animals in the testing set from the animals assigned to the training population. Because of this, a multiple-step genomic evaluation was performed. In the first step, we fitted an animal model that included pedigree-based random additive genetic effects (i.e. EBV), CG fixed effects, and the linear effect of the animal’s age at slaughter. For REA, HCW was also added to the model as a covariate to correct for the size of the animal. Single-trait analyses were performed for all traits, except for HCW. In this case, weaning weight was used as an anchor trait in a bivariate analysis to correct for the effect of selection for this trait. The analyses were performed by Bayesian inference using the BLUPF90 family programs [19]. A total of 1,000,000 Gibbs samples were generated, considering a burn-in period of 100,000 iterations with samples taken every 50 cycles. Variance components and heritability estimates are in Table 1.

**Table 1:** Variance components and heritability estimates for each trait

Trait	Estimates		
	$\varvec{\sigma}_{{\mathbf{a}}}^{2}$	$\varvec{\sigma}_{\varvec{e}}^{2}$	h^2^ (SD)
Rib eye area (cm^2^)	10.88	41.71	0.20 (0.10)
Backfat thickness (mm)	0.30	3.31	0.08 (0.06)
Hot carcass weight (kg)	47.80	238.25	0.17 (0.07)

$\sigma_{a}^{2}$ additive genetic variance, $\sigma_{e}^{2}$ residual variance, *h* ^*2*^ heritability, *SD* standard deviation of the heritability estimates

From these analyses, two alternative quantities were computed and used as response variables for genomic prediction in the second step i.e. estimated breeding value (EBV) and phenotype adjusted for fixed effects (Y*). The corresponding EBV presented average accuracies equal to 0.47, 0.31 and 0.43 for REA, BFT and HCW, respectively. Accuracies of EBV were obtained using the following formula:

$${\text{acc }} = \, \surd \left( {1 - {\text{PEV}}/{{\upsigma }}_{\text{g}}^{ 2} } \right) ,$$

where PEV is the prediction error variance, and ${{\upsigma }}_{\text{g}}^{ 2}$ is the additive genetic variance of the trait. The descriptive statistics of the response variables are summarized in Table 2.

**Table 2:** Descriptive statistics of the pseudo-phenotypes for rib eye area (REA), backfat thickness (BFT), and hot carcass weight (HCW)

Trait	N	Types	Mean	SD	Minimum	Maximum
REA (cm^2^)	1567	Y*	−0.08	6.90	−24.72	27.96
		EBV	−0.03	1.50	−5.50	5.79
BFT (mm)	1566	Y*	0.02	1.82	−7.51	11.48
		EBV	0.01	0.16	−0.65	0.94
HCW (kg)	1409	Y*	0.24	16.23	−65.33	66.44
		EBV	0.15	2.87	−10.81	10.22

Y* phenotype adjusted for fixed effects, *EBV* estimated breeding value, *N* number of animals with phenotypes, *SD* standard deviation

The choice of using EBV rather than deregressed EBV (dEBV) as response variable was based on the fact that available EBV had been predicted with low accuracy. According to [15], dEBV obtained under such conditions are expected to incorporate too much noise during the deregression process, in which parental contribution is removed. Thus, EBV were considered to be a better option for genomic prediction in this scenario [9].

### Genomic predictions

Genomic predictors for both Y* and EBV were obtained using the Bayesian generalized linear regression (BGLR) package [20], considering three models: Bayesian ridge regression (BRR), in which independent normal distributions with homogeneous variance are assumed as prior distributions of the SNP effects; Bayes C (BC), in which the prior probabilities of the SNP effects consist of a mixture of a probability mass point at zero (p = 1−π) and a Gaussian distribution (p = π); and Bayesian Lasso (BL) in which a double exponential distribution is assumed as a prior distribution of SNP effects.

The general model for genomic prediction was expressed in matrix notation as follows:

$${\mathbf{y}} = {\mathbf{1}}\mu + {\mathbf{Wg}} + {\mathbf{e}} ,$$

where **y** is the vector of pseudo-phenotypes (Y* or EBV), *μ* is the overall mean, **g** is the vector of marker effects, **W** contains the genotype (coded as 0 = AA, 1 = AB or 2 = BB) for each individual and each marker, and **e** is the vector of residual effects. The prior distribution assigned to **g** differs depending on the model (BRR, BC or BL), as explained in the next sections.

In the case of BRR, independent normal distributions with zero mean and homogeneous variance $\sigma_{\beta }^{2}$ were assigned to the marker-specific regression coefficients. Furthermore, a scaled inverse Chi squared distribution (${\mathcal{X}}^{ - 2}$) was assigned as prior distribution for $\sigma_{\beta }^{2}$, with hyperparameters $\text{d}f_{\beta }$ and $S_{\beta }$. The number of degrees of freedom ($\text{d}f_{\beta }$) was equal to five and the scale parameter ($S_{\beta }$) was considered as a function of raw measurements of the observed dispersion in the sample and of a prior R^2^ assigned to the model. By default, the BGLR package attributes a value of 0.5 to R^2^, which means that the values of the hyperparameters are determined so that 50 % of the variability is attributed a priori to the linear predictors and 50 % to the residuals of the model [20].

The prior distribution of SNP effects in BC is similar to that in BRR, but in addition, it considers a parameter π which expresses the proportion of SNPs with non-null effects. This parameter is treated as unknown and has a beta density function a priori, i.e. π ~ *beta*(p_0_, π_0_), with parameter spaces p_0_ > 0 and π_0_ ∊ [0, 1]. It was assumed that π_0_ was equal to 0.5 and p_0_ was equal to 10 [20].

For BL, the marginal prior distribution for each SNP effect is a double exponential function [18, 20], which includes a parameter *λ*^2^ that was treated as unknown, with a prior distribution *λ*^2^ ~ gamma(*r*, *s*). The BGLR package considers by default that *r* = 1.1 and calculates the scale parameter *s* based on the “prior” R^2^ of the model, as for BRR [20].

In the analyses including EBV as the response variable, the respective accuracies that were computed in the first step were used as weighting factors [15]. When Y* was used as response variable, weighting factors were calculated according to [18] as follows: the weight of the phenotype of the *i*th animal was the square root of the diagonal of the (co) variance matrix of the adjusted residuals **Σ**, in such a way that **Σ** = (**I**−**H**)cov(y)(**I**−**H**′), where **I** is an identity matrix and **H** is the so-called hat matrix, given by **H** = **X**[**X**′**V**^−1^**X**]^−1^**X**′**V**^−1^, with **X** corresponding to the incidence matrix of fixed effects. In addition, **V** = **ZGZ**′*’* + **R**, where **G** and **R** are diagonal matrices of additive genetic and residual variances, respectively, and **Z** is the incidence matrix that links observations to their respective animal random effect.

For all models, the predicted DGV were computed using the following equation:

$$DGV_{i} = \sum\limits_{j = 1}^{p} {w_{ij} \hat{g}_{j} } ,$$

where *p* is the number of SNPs; *w*_*ij*_ is the genotype of animal *i* for SNP *j* (coded as 0, 1 or 2), and *ĝ*_*j*_ is the estimated SNP substitution effect for SNP *j* that was estimated from the training population.

The prediction ability of DGV was evaluated by cross-validation. The training and test datasets were generated completely at random from the reference population, so that the cross-validation scheme could better mimic how these prediction equations could be used in practice.

Before analysis, the animals were randomly divided into five groups so that a 5-fold cross-validation scheme was applied. More specifically, data from four groups (training dataset) were used to fit the models and the prediction quality resulting from the inferred marker effects was evaluated using the genotypes and response variables of the remaining group (validation or test dataset). Each model was fitted five times, each time treating a different group as the validation set. The prediction quality attributed to each model was based on the average performance obtained for the validation set in the five evaluations. For REA and BFT, the average sizes of training and validation populations were equal to 1255 and 314, respectively, and for HCW, they were equal to 1129 and 282, respectively.

### Criteria for the comparison of models

Correlation and regression coefficients between response variable (Y* or EBV) and DGV, and the mean-squared error (MSE) of predictions, were used to evaluate and compare the prediction ability of the models. When EBV was used as the pseudo-phenotype, the simple correlation of this quantity with the computed DGV was considered as an empirical measure of accuracy [9, 15]. On the one hand, in the analyses that used Y* as response variable, prediction accuracy was obtained by dividing the correlation between Y* and DGV by the square root of the heritability *h* of the trait [6, 21, 22]. This is an approximation of the correlation between DGV and true breeding value (TBV), which corresponds to true accuracy [6, 22]. On the other hand, when EBV is used as the pseudo-phenotype, the correlation between EBV and DGV may be seen as an upper limit of the correlation between TBV and DGV [23], which already corresponds to a non-biased estimate of accuracy [9, 15, 23].

The linear regression coefficient of the pseudo-phenotype on DGV was considered to express the magnitude of inflation/deflation of DGV relative to the response variable. Values close to one are considered the most desirable. The MSE was also used as a measure of the prediction ability of the models, which combines quality assessment in terms of variance and bias of predictions.

### Genomic relationships between training and validation datasets

Since the degree of relationships between training and validation datasets influences prediction ability [24, 25], the relationship between these two groups was estimated using the genomic relationship matrix **G**, computed as described by [17]. We examined the following features of this matrix **G**: maximum genomic relationship between each animal of the validation set and all animals of the training set (maxr); and average of the five (mean5) and ten (mean10) highest relationship values between each animal in the validation with all the animals in the training set. These relationship statistics were consequently averaged across all animals and across all folds.

## Results and discussion

The existence of LD between SNPs and QTL is an essential assumption in the use of SNPs to predict the genetic merit of animals [5]. Since the localization of a QTL is generally unknown, the level of LD between adjacent markers is determined as an indicator of the plausibility of this assumption [8]. Previous studies in Nellore cattle using the bovine high-density SNP panel (777K) reported average values of LD, measured by the r^2^ statistic, equal to 0.17 [26] and 0.29 [10], which were considered sufficiently high to perform genomic predictions. Using the same panel, we found an average LD of 0.31.

In general, the predicted accuracies for each trait were practically the same for the three models used in this study (Table 3), in agreement with the literature [18, 27]. According to [18], studies using real data do not always reveal relevant differences between models, which can be attributed to the large number of regression coefficients that need to be inferred from a small number of samples (n <<< p). In such a situation, insufficient information from the data restricts the Bayesian learning process. As a consequence, although the Bayesian models may produce quite different inferences regarding individual SNP effects, they often result in similar predictive abilities in cross-validation checks [12]. Another factor that could explain the similarity of results across models is the complex nature of the traits studied. According to [28], different models tend to show similar predictive ability when the traits are affected by many small-effect loci.

**Table 3:** Empirical prediction accuracies measured by Pearson’s correlation between pseudo-phenotype and direct genomic breeding values [r(y_i_,DGV)] and standard deviation (SD) for rib eye area (REA), backfat thickness (BF) and hot carcass weight (HCW) obtained with different models and the average of 5-fold cross-validation

Trait	Type	r(y_i_,DGV)^a^ ± SD		
		BRR	BC	BL
REA (cm^2^)	Y*	0.46 ± 0.056	0.46 ± 0.057	0.47 ± 0.056
	EBV	0.36 ± 0.057	0.35 ± 0.057	0.36 ± 0.059
BFT (mm)	Y*	0.21 ± 0.029	0.23 ± 0.031	0.22 ± 0.029
	EBV	0.25 ± 0.026	0.25 ± 0.027	0.25 ± 0.025
HCW (kg)	Y*	0.37 ± 0.053	0.36 ± 0.058	0.37 ± 0.056
	EBV	0.33 ± 0.041	0.33 ± 0.044	0.33 ± 0.043

^a^For the Y* as response variable, r(y_i_,DGV) was divided by the square root of heritability of the trait

Y* phenotype adjusted for fixed effects, *EBV* estimated breeding value, *y*_*i*_ pseudo-phenotype, *BRR* bayesian ridge regression, *BC* Bayes C, *BL* Bayesian Lasso

Linear regression coefficients [*b*(y*, DGV)] and MSE were also similar across models (Table 4). The smallest regression coefficient was observed for BFT preadjusted for fixed effects, which is consistent with its low heritability estimate (0.08). In this case, the fit of the regression model may have been affected by its higher noise to signal ratio.

**Table 4:** Regression coefficient of the pseudo-phenotype on direct genomic breeding values [b(y_i_,DGV)] and mean squared error of prediction (MSE) for rib eye area (REA), backfat thickness (BFT) and hot carcass weight (HCW) obtained with different models to estimate SNP effects

Trait	Type	b(y_i_,DGV)			MSE		
		BRR	BC	BL	BRR	BC	BL
REA (cm^2^)	Y*	0.99	0.93	1.02	45.56	45.62	45.56
	EBV	1.07	1.02	1.09	1.95	1.96	1.96
BFT (mm)	Y*	0.40	0.37	0.39	3.30	3.33	3.32
	EBV	0.90	0.90	0.98	0.03	0.03	0.03
HCW (kg)	Y*	0.93	0.80	0.96	261.5	262.0	261.8
	EBV	1.11	1.07	1.14	7.35	7.37	7.36

Y* phenotype adjusted for fixed effects, *EBV* estimated breeding value, *y*_*i*_ pseudo-phenotype, *BRR* bayesian ridge regression, *BC* Bayes C, *BL* Bayesian Lasso, *REA* rib eye area, *BFT* backfat thickness, *HCW* hot carcass weight, ${\text{MSE }} = \frac{1}{N}\mathop \sum \nolimits (y_{i} - DGV)^{2}$

While the three models provided similar results, the use of different types of pseudo-phenotypes produced different prediction accuracies (Table 3), which agrees with the literature [9, 15]. The variables Y* and EBV are essentially distinct quantities and thus, their ratios between genetic signal and noise differ [28]. As a consequence, the correlation between the DGV derived from each one of these variables and the TBV tend to differ as well. This difference, which might represent an advantage for either one of these response variables, depends on the dataset used and the specific application [15].

The empirical accuracies of predictions based on EBV were 23 % (REA) and 10 % (HCW) lower than the predictions based on adjusted phenotype. These results indicate that using adjusted phenotype instead of EBV as the pseudo-phenotype in genomic prediction is an advantage for more highly heritable traits. For BFT, which was the least heritable (0.08 vs. 0.20 and 0.17 for REA and HCW, respectively), prediction accuracy was greater when EBV was used as the response variable.

In our study, the relatively small dataset and the low heritability of the traits analyzed limited the accuracies of EBV, which ranged from low to moderate. This may have contributed to weakening the prediction abilities observed here. According to [7], scenarios that involve highly heritable traits and large numbers of phenotypic records and genotyped animals can certainly lead to higher accuracies of DGV.

The accuracies of DGV obtained for Nellore cattle in this study were greater than those reported for Angus (0.16), Shorthorn (0.19), Brahman (0.28) and Santa Gertrudis (0.29) cattle, and were similar to those reported for Hereford (0.32), Belmont Red (0.33) and Murray Grey (0.39) cattle for carcass weight [7]. Similar accuracies of DGV were also reported in [29] i.e. 0.35 (Angus) and 0.33 (Charolais) for carcass weight, 0.36 (Angus) and 0.24 (Charolais) for REA, and 0.33 (Angus) and 0.46 (Charolais) for carcass average BFT. In both studies [7, 29], accuracies of DGV were calculated as the correlation between adjusted phenotypic values and DGV, divided by the square root of the heritability. For Nellore cattle, accuracies of genomic prediction have been reported only for growth, reproductive and visual score traits [10] with values ranging from 0.17 (navel at weaning) to 0.74 (finishing precocity) using deregressed EBV as response variable.

It is known that the cross-validation strategy may change estimates of accuracy. There are already a few articles in the literature that show that stronger genetic ties between animals in the training and test sets improve accuracies [24, 25]. Different cross-validation strategies can be used, for example, to assess a best and worst case scenarios. However, a general recommendation is that the final cross-validation design reflects how genomic predictions will be used in practice for that specific species, breed and application [21]. For example, animals could be sampled at random to build the training and test sets, if the SNP chip is to be used for commercial herds, with different genetic relationships between selection candidates and the reference population.

In general, accuracies of DGV reported for various traits in beef cattle support the feasibility of applying genomic selection [7, 10, 15, 21, 29] and different methodologies are available to perform genomic prediction [30]. However, in practice, this technology is still rarely applied in the beef cattle industry especially for phenotypes that are difficult or expensive to measure, such as carcass traits, which is probably due to the lack of sufficiently large reference populations [4, 7]. Van Eenennaam et al. [4] raised several issues related to the beef cattle industry that hamper the implementation of genomic selection. Among these, they listed the segmented nature of this sector in terms of uniformity of breeding goals, the importance of crossbreeding in meat production systems, and the limited use of assisted reproduction techniques. These authors also highlighted that, for beef cattle, research groups work in a somewhat isolated manner, which restricts the availability of reference populations for each group in terms of scope and results. Furthermore, beef cattle populations consist mainly of various breeds and/or mixture of breeds, which makes it more difficult and also less useful to establish combined reference populations. The number of individuals of a single breed in a reference population is often small and thus, it becomes difficult to accurately identify the additive differences among breeds in genomic prediction of crossbred animals [31].

The fact that the Nellore is the dominant breed in the Brazilian beef cattle farming might be an advantage for the adoption of genomic selection, since it may be relatively easier to create reference populations of sufficient size. However, this would require pooling the various datasets that have been created concomitantly in Brazil by independent groups [1].

Although accuracies of genomic predictions tend to increase with the size of the reference population [7, 15], other factors can also affect accuracies. Some of these factors are the heritability of the trait (or the accuracy of the pseudo-phenotype), the density of the SNP panel and level of LD, the degree of relationship between training and validation animals, genetic architecture of the trait, and the model used [32]. Since SNPs capture both LD and additive genetic relationships [4, 24], a decrease in the level of relationship reduces accuracy of prediction [28]. We used a dataset that was structured in half-sib families. As expected, the average of the maximum relationship (maxr) was equal to about 0.25 whereas the mean5 and mean10 statistics were equal to 0.19 and 0.17, respectively. These values indicate that the degree of relationship between training and validation populations was relatively low, taking as a benchmark the study of [24]. In [24], three scenarios with varying magnitudes of the relationship between reference and validation populations: (1) close relationships (when the animals of the validation population had 20 half-sibs in the training population), (2) distant relationships (no close relationships between individuals in the training and testing populations except for a few second degree relationships, i.e. cousins), and (3) no relationship. In our study, the mean5 and mean10 statistics had values that were similar to those of scenario (2). This finding suggests that the accuracy of prediction obtained in this population, besides capturing the relationship between individuals of the two groups, is also due to the LD between markers and QTL [24]. The results of this research can likely be extrapolated to real breeding programs for Nellore since a variety of commercial herds that had industry-relevant genetic backgrounds were represented.

## Conclusions

We showed that applying genomic selection to improve carcass traits in breeding programs of Nellore cattle is feasible since moderate genomic prediction accuracies can be achieved. Prediction of genomic breeding values from adjusted phenotypes Y* was more accurate than from EBV, especially for highly heritable traits. The three genomic prediction models considered (BRR, BC and BL) presented similar predictive performances and thus, they could be equally recommended for the implementation of genomic selection for carcass traits in Nellore cattle.
